# Standardized postpartum blood pressure protocol and lower rates of hypertension at 6 weeks postpartum

**DOI:** 10.1002/pmf2.70477

**Published:** 2026-10-08

**Authors:** Audra C. Fain, Thalia Mok, Jenny Y. Mei

**Affiliations:** ^1^ Division of Maternal‐Fetal Medicine Department of Obstetrics and Gynecology University of California Los Angeles USA

**Keywords:** chronic hypertension, gestational hypertension, labetalol, nifedipine, obstetrics, postpartum, postpartum hypertension, preeclampsia, standardized clinical action management plan

## Abstract

**Background:**

Hypertensive disorders of pregnancy (HDP) increase the risk of chronic hypertension (HTN) and cardiovascular (CV) disease later in life. It is unknown how strict blood pressure (BP) monitoring postpartum (PP) may affect rates of HTN at 6 weeks PP.

**Objective:**

We evaluate the effect of a PP HTN standardized clinical assessment and management plan (SCAMP) on HTN at 6 weeks PP.

**Study design:**

This is a secondary analysis of a prospective cohort study of patients with PP HTN at a quaternary care center for 6 months after enacting an institution‐wide SCAMP. The prospective group was compared with historical controls who delivered prior to initiation of the SCAMP. The SCAMP included (1) initiation or uptitration of medication for any BP ≥150/100 or any 2 BPs ≥140/90 within a 24‐h period with a goal of BP <140/90 12 h prior to discharge and (2) enrollment in a remote BP monitoring system upon discharge. Inclusion criteria were an existing diagnosis of chronic HTN or HDP and a documented BP at a 6‐week PP visit. The primary outcome was HTN, defined as systolic BP (SBP) ≥140 or diastolic BP (DBP) ≥90, at 6 weeks PP. Secondary outcomes included mean BP values as measured in the outpatient setting at 6 weeks PP and use of anti‐HTNs at the time of the 6‐week PP visit. For all analyses, *p* values were two‐way, and the level of statistical significance was set at *p* < 0.05.

**Results:**

Of 780 PP HTN patients in the prospective cohort study, 627 (80.4%) met inclusion criteria for the secondary analysis, 305 in the SCAMP cohort and 322 historical controls. The SCAMP cohort had lower rates of chronic HTN, more patients on anti‐HTNs, and higher rate of nifedipine use. The primary outcome of HTN at 6 weeks PP was significantly lower in the SCAMP group (10.5% vs. 18.3%, *p* = 0.005). After adjusting for confounding variables, multivariable logistic regression showed that the SCAMP group had 38% reduced odds of HTN at 6 weeks PP compared to controls (adjusted odds ratio [aOR], 0.62; 95% confidence interval [CI], 0.38–0.996; *p* = 0.048). Mean SBP at 6 weeks PP was significantly lower in the SCAMP group (120 ± 12 vs. 124 ± 14 mmHg, *p* < 0.001), as was mean DBP (76 ± 8 vs. 81 ± 8 mmHg, *p* < 0.001). A subanalysis only including patients with diagnosed HDP and excluding those with only chronic HTN demonstrated similar findings.

**Conclusion:**

Implementation of a SCAMP was associated with reduced rates of HTN at 6 weeks PP, with lower mean SBP and DBP. More data are needed to evaluate how these findings mitigate long‐term HTN risk.

## 1 INTRODUCTION

Postpartum (PP) hypertension (HTN) is a leading cause of maternal morbidity. Hypertensive disorders of pregnancy (HDP), including gestational HTN and preeclampsia, complicate approximately 10% of all pregnancies in the United States [1, 2, 3], making PP HTN a primary focus of quality improvement initiatives [4, 5, 6]. Given the substantial cardiovascular (CV) risks during pregnancy and PP, effective PP management of HTN is critical for maternal health [7, 8, 9].

The relationship between HDP and long‐term CV health has become increasingly apparent in recent years. Women with a history of gestational HTN or preeclampsia face significantly elevated risks of CV disease in the decades following pregnancy [10]. HDP are associated with a 2‐fold higher risk of coronary heart disease, cerebrovascular disease, peripheral arterial disease, and CV mortality compared to women with normotensive pregnancies [2, 10]. Elevations in PP BP at 6 weeks in patients with HDP have been associated with increased BP over the following 4–13 years of life [11, 12]. Despite this relationship, there is no standardized approach for PP blood pressure (BP) management [13, 14, 15, 16, 17].

Standardized BP protocols incorporating remote BP monitoring and structured anti‐HTN initiation protocols have emerged as a promising intervention to improve PP HTN and reduce readmission [1, 18, 19, 20, 21, 22, 23]. While reduction of PP readmissions is an important short‐term outcome, it is currently unclear whether tighter BP control in the immediate PP period impacts rates of HTN at 6 weeks PP.

We evaluated whether implementation of a PP HTN standardized clinical assessment and management plan (SCAMP) was associated with reduced HTN at 6 weeks PP, defined as systolic BP (SBP) ≥140 mmHg or diastolic BP (DBP) ≥90 mmHg. Secondary objectives included comparison of mean BP values, timing of BP ascertainment, and use of anti‐HTN medications between women managed under the SCAMP and historical controls. We hypothesized that implementation of the SCAMP would be associated with lower rates of HTN at 6 weeks PP, reflecting better overall BP control and potentially improved long‐term rates of HTN.

## 2 METHODS

We conducted a secondary analysis of a prospective cohort study of patients with HDP and chronic HTN at a single tertiary care center. The primary analysis evaluated the effect of a PP HTN SCAMP on readmissions and emergency department (ED) visits [1]. This secondary analysis specifically examined the effect of the SCAMP on HTN measurement at 6 weeks PP.

### 2.1 Study design and setting

We performed a secondary analysis comparing patients with chronic HTN or HDP who delivered at an academic quaternary care center during the 6‐month period following institution‐wide SCAMP initiation in April 2022 (postintervention group) with a historical control group who delivered at the same center during the 6 months prior to April 2022. The diagnosis of chronic HTN or HDP was determined by delivery admission discharge diagnoses. Prior to SCAMP initiation, PP BP management was individualized by the provider on service that day. There were no institutional guidelines for the length of time normotensive prior to discharge, and PP monitoring typically consisted of a 1‐week BP check in clinic. Data collection for the postintervention group started 1 month after implementation of the SCAMP to allow for the transition to a new intervention. The postintervention cohort included eligible patients delivering between April 2022 and October 2022, while the historical control group included patients delivering October 2021 through March 2022.

### 2.2 Participants

For this secondary analysis, patients were eligible if they had delivered during the specified study time range, were age 18 years or older, and had a diagnosis of chronic HTN or an HDP prior to discharge from their delivery admission. We only included patients who had a documented BP at an outpatient visit 4–8 weeks after delivery. Patients in the historical control group were identified through retrospective electronic medical record review of all patients delivering during the 6 months before implementation of the SCAMP. Patients were excluded if they did not have a hypertensive disease diagnosis before their delivery admission discharge, had previable deliveries (before 24 weeks of gestation), had a stillbirth, or did not have a documented PP BP during the time frame.

### 2.3 SCAMP intervention

The SCAMP was introduced to providers at the study site in April 2022, with monthly reminders on the SCAMP guidelines sent throughout the study period. The SCAMP included both inpatient and outpatient components.

Inpatient recommendations included PP initiation or uptitration of medication for any BP higher than 150/100 mmHg or any two BPs higher than 140/90 mmHg within a 24‐h period, with the goal of achieving normotension (BP lower than 140/90 mmHg) in the 12 h before discharge. The on‐call physician team determined anti‐HTN medication and dosing to meet discharge criteria. Prior to discharge, all patients with a diagnosis of HTN were provided a standardized hospital BP cuff and instructions.

Outpatient follow‐up included a remote BP monitoring system linked to the patient's mobile electronic medical record (EMR) application. Patients logged BPs twice daily through the application, with abnormal values both notifying a physician on the study team and prompting the patient to reach out to their provider. Patients were prompted twice daily to input BPs into the application. Patients were contacted within 24 h of inputting abnormal BPs, defined as SBP >140 or DBP >90, and medication adjustments were made per the SCAMP protocol. Medication regimen at the time of discharge was documented, and timing of uptitration or discontinuation of anti‐HTN was noted. Medication compliance was based on patient report. All patients who were on anti‐HTN on the day of the 6‐week PP visit, including those who were then discontinued off anti‐HTN during the 6‐week visit, were counted as being on anti‐HTN at 6 weeks. The remote BP monitoring program stopped for all patients at 6 weeks PP. Additional details can be found in the primary analysis.

### 2.4 Data collection

IRB‐approved study members abstracted maternal demographic data and obstetric outcome data from the EMR. Race and ethnicity were self‐classified by the patient according to options defined by the study institution. Characteristics of the perinatal course were also abstracted, including hypertensive diagnosis, mode of delivery, first occurrence of BP in the EMR following discharge, and timing of anti‐HTN medication initiation.

### 2.5 Outcomes

The primary outcome for this secondary analysis was HTN at 6 weeks PP, defined as SBP ≥140 mmHg or DBP ≥90 mmHg based on a single BP reading obtained at the PP visit. For the purposes of simplicity in our paper, we are using the term ‘hypertension’ to define any elevated BP ≥ 140/90, but it does not indicate persistent or chronic hypertension. PP visits were defined as any visit occurring between 4–8 weeks after delivery. If patients had multiple visits within this window, the visit closest to 6 weeks PP was used.

Secondary outcomes included: (1) mean SBP and DBP values at the 6‐week PP visit; (2) presence of any BP recorded prior to the 6‐week appointment (defined as any documented BP, including remote monitoring logs, clinic visits, ED visits, or other documented measurements in the medical record); (3) timing of first BP ascertainment (number of days from discharge to first documented BP); (4) use of anti‐HTN medications at 6 weeks PP; (5) continued use of anti‐HTN medications beyond 6 weeks PP; (6) referral to or visit with primary care physician or cardiologist during the PP period; and (7) PP readmission for HTN.

We also ran a subanalysis excluding patients with only chronic hypertension and thus only including patients with hypertensive diseases of pregnancy. This was aimed to help mitigate the potential confounding impact of chronic hypertension on long term hypertension outcomes.

### 2.6 Statistical analysis

Sample size for this secondary analysis was determined by the number of patients in the primary cohort study who had documented 6‐week PP visit data. As this was a secondary analysis, no a priori power calculation was performed specifically for the HTN outcome.

Descriptive statistics were used to characterize baseline demographics and clinical characteristics. Continuous variables were reported as mean ± standard deviation or median (interquartile range), as appropriate. Categorical variables were reported as frequencies and percentages. Independent‐sample *t*‐tests, Mann–Whitney U tests, and chi‐square tests were used to compare baseline characteristics and outcomes between the postintervention and historical control groups as appropriate.

Multivariable logistic regression was performed to evaluate the association between SCAMP implementation and HTN at 6 weeks PP, controlling for potential confounders. Variables included in the model were selected a priori based on clinical relevance and included chronic HTN, aspirin use during pregnancy, anti‐HTN medication prescribed on discharge, and type of anti‐HTN medication prescribed (nifedipine, labetalol, or both).

Results of logistic regression were reported as adjusted odds ratios (aOR) with 95% confidence intervals (CI). For all analyses, *p* values were two‐sided, and the level of statistical significance was set at *p* < 0.05. Statistical analyses were performed using SPSS 21. This research was approved by the institutional IRB.

## 3 RESULTS

Of the 780 patients included in the primary prospective cohort study, 627 (80.4%) met inclusion criteria for this secondary analysis, with 305 patients in the postintervention (SCAMP) group and 322 historical controls (Figure 1). Baseline demographics were overall similar between groups, though the SCAMP group had a lower prevalence of chronic HTN (23.3% vs. 32.9%, *p* = 0.007) and a higher proportion of patients discharged on anti‐HTN medications (33.1% vs. 23.3%, *p* = 0.006) (Table 1). There were also higher rates of nifedipine use in the SCAMP cohort (65% vs. 42.7%, *p* = 0.001).

**FIGURE 1:**
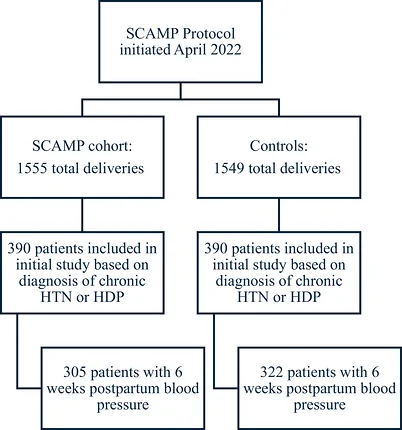
Patient inclusion flowchart. HDP, hypertensive disorders of pregnancy; HTN, hypertension; SCAMP, standardized clinical assessment and management plan.

**TABLE 1:** Baseline characteristics between groups.

Maternal demographics	SCAMP (*n* = 305)	Controls (*n* = 322)	*p* value
Maternal age (years, mean ± SD)	34.2 ± 5.17	34.7 ± 4.79	0.18
Maternal age 35–39	151 (49.5%)	171 (53.1%)	0.37
Maternal age ≥ 40	40 (13.1%)	47 (14.6%)	0.59
Race/Ethnicity			
White	130 (42.6%)	145 (45.0%)	0.67
Black	34 (11.1%)	33 (10.2%)	
Hispanic/Latina	57 (18.7%)	70 (21.7%)	
Asian/Pacific Islander	60 (19.7%)	52 (16.1%)	
Other	24 (7.9%)	22 (6.8%)	
Gravida (median, IQR)	2 (1,2)	2 (1,3)	0.5
Parity (median, IQR)	1 (1,2)	1 (1,2)	0.51
Nulliparity	215 (70.5%)	221 (68.6%)	0.61
BMI at delivery (kg/m^2^, mean ± SD)	29.0 ± 5.6	28.1 ± 6.9	0.08
Gestational age (mean ± SD)	38w2d ± 15d	38w2d ± 15d	0.62
Preterm delivery	44 (14.4%)	42 (13.0%)	0.62
Chronic hypertension	71 (23.3%)	106 (32.9%)	**0.007**
Pregestational diabetes mellitus	9 (3.0%)	9 (2.8%)	0.91
Gestational diabetes	27 (8.9%)	34 (10.6%)	0.47
Autoimmune disease	5 (1.6%)	2 (0.6%)	0.23
Renal disease	2 (0.7%)	6 (1.9%)	0.18
Multifetal gestation	11 (3.6%)	6 (1.9%)	0.18
Aspirin use	91 (29.8%)	134 (41.6%)	**0.002**
In vitro fertilization	30 (9.8%)	37 (11.5%)	0.5
Insurance type			
Private	268 (87.9%)	290 (90.1%)	0.38
Public or None	37 (12.1%)	32 (9.9%)	
Hypertensive diagnosis at discharge			
Gestational hypertension	132 (43.3%)	130 (40.4%)	0.064
Preeclampsia without severe features	61 (20.0%)	48 (14.9%)	
Preeclampsia with severe features	63 (20.7%)	65 (20.2%)	
HELLP	1 (0.3%)	4 (1.2%)	
Chronic hypertension only	48 (15.7%)	75 (23.3%)	
Proteinuria	101 (33.1%)	94 (29.2%)	0.29
Magnesium sulfate given	64 (21.0%)	68 (21.1%)	0.97
IV anti‐HTN given	33 (10.8%)	40 (12.4%)	0.53
Method of diagnosis of severe preeclampsia			
Severe range blood pressures	43 / 63 (68.3%)	49 / 68 (72.1%)	0.89
Lab abnormalities	19 / 63 (30.2%)	18 / 68 (26.5%)	
Clinical symptoms	1 / 63 (1.6%)	1 / 68 (1.5%)	
Anti‐HTN prescribed on discharge	101 (33.1%)	75 (23.3%)	**0.006**
Type of anti‐HTN prescribed			
Nifedipine	65 / 100 (65%)	32 / 75 (42.7%)	**0.001**
Labetalol	16 / 100 (16%)	30 / 75 (40%)	
Both	19 / 100 (19%)	13 / 75 (17.3%)	

*Note*: Data presented as *n* (%) unless otherwise indicated. *p* value calculated for *t*‐test, Mann–Whitney *U*, and chi‐square as appropriate to compare groups. *p* value significance was set at < 0.05 and significant values are bolded. Logistic regression was performed controlling for chronic HTN, aspirin use, anti‐HTN medication on discharge, and type of anti‐HTN medication.

Abbreviations: anti‐HTN, anti‐hypertensives; BMI, body mass index; HELLP, hemolysis elevated liver enzymes low platelets; IQR, interquartile range; IV, intravenous; SCAMP, standardized clinical assessment and management plan; SD, standard deviation.

The primary outcome of HTN at 6 weeks PP was significantly lower in the SCAMP group at 10.5% compared with 18.3% in the historical control group (*p* = 0.005) (Table 2, Figure 2). After adjusting for chronic HTN, aspirin use, anti‐HTN medication prescribed on discharge, and type of anti‐HTN, the SCAMP group had 38% reduced odds of HTN at 6 weeks PP compared to controls (adjusted odds ratio [aOR], 0.62; 95% confidence interval [CI], 0.38–0.996; *p* = 0.048).

**TABLE 2:** Six‐week postpartum hypertension outcomes between groups.

Six‐week postpartum outcomes	SCAMP (*n* = 305)	Controls (*n* = 322)	*p* value	Adjusted odds ratio (95% CI)
HTN at 6 weeks PP (SBP ≥ 140 or DBP ≥ 90)	32 (10.5%)	59 (18.3%)	**0.005**	**0.62 (0.38–0.996)**
Average systolic BP at 6 weeks PP (mmHg, mean ± SD)	120 ± 12	124 ± 14	**<0.001**	N/A
Average diastolic BP at 6 weeks PP (mmHg, mean ± SD)	76 ± 8	81 ± 8	**<0.001**	N/A
Any BP recorded prior to 6‐week appointment	268 (87.9%)	200 (62.1%)	**<0.001**	**4.35 (2.86–6.62)**
Number of days after discharge of first BP ascertainment (days, median, IQR)	1 (1,6)	13 (3, 41)	**<0.001**	N/A
Postpartum readmission	5 (1.6%)	23 (7.1%)	**0.001**	**0.22 (0.08–0.58)**
On anti‐HTN medication at 6 weeks PP	49 (16.1%)	58 (18.0%)	0.52	0.73 (0.43–1.22)
Continued on anti‐HTN medication past 6 weeks PP	44 (14.4%)	49 (15.2%)	0.78	0.92 (0.53–1.61)
Seen by primary care or cardiology PP	152 (49.8%)	186 (58.1%)	**0.038**	**0.72 (0.52–0.99)**

*Note*: Data presented as *n* (%) unless otherwise indicated. *p* value calculated for *t*‐test, Mann–Whitney U, and chi‐square as appropriate to compare groups. *p* value significance was set at < 0.05 and significant values are bolded. Logistic regression was performed controlling for chronic HTN, aspirin use, anti‐HTN medication on discharge, and type of anti‐HTN medication. Patients were included as seen by primary care or cardiology if they attended an outpatient visit, either virtually or in‐person, within 12 weeks postpartum.

Abbreviations: BP, blood pressure; DBP, diastolic blood pressure; HTN, hypertension; IQR, interquartile range; PP, postpartum; SBP, systolic blood pressure; SCAMP, standardized clinical assessment and management plan.

**FIGURE 2:**
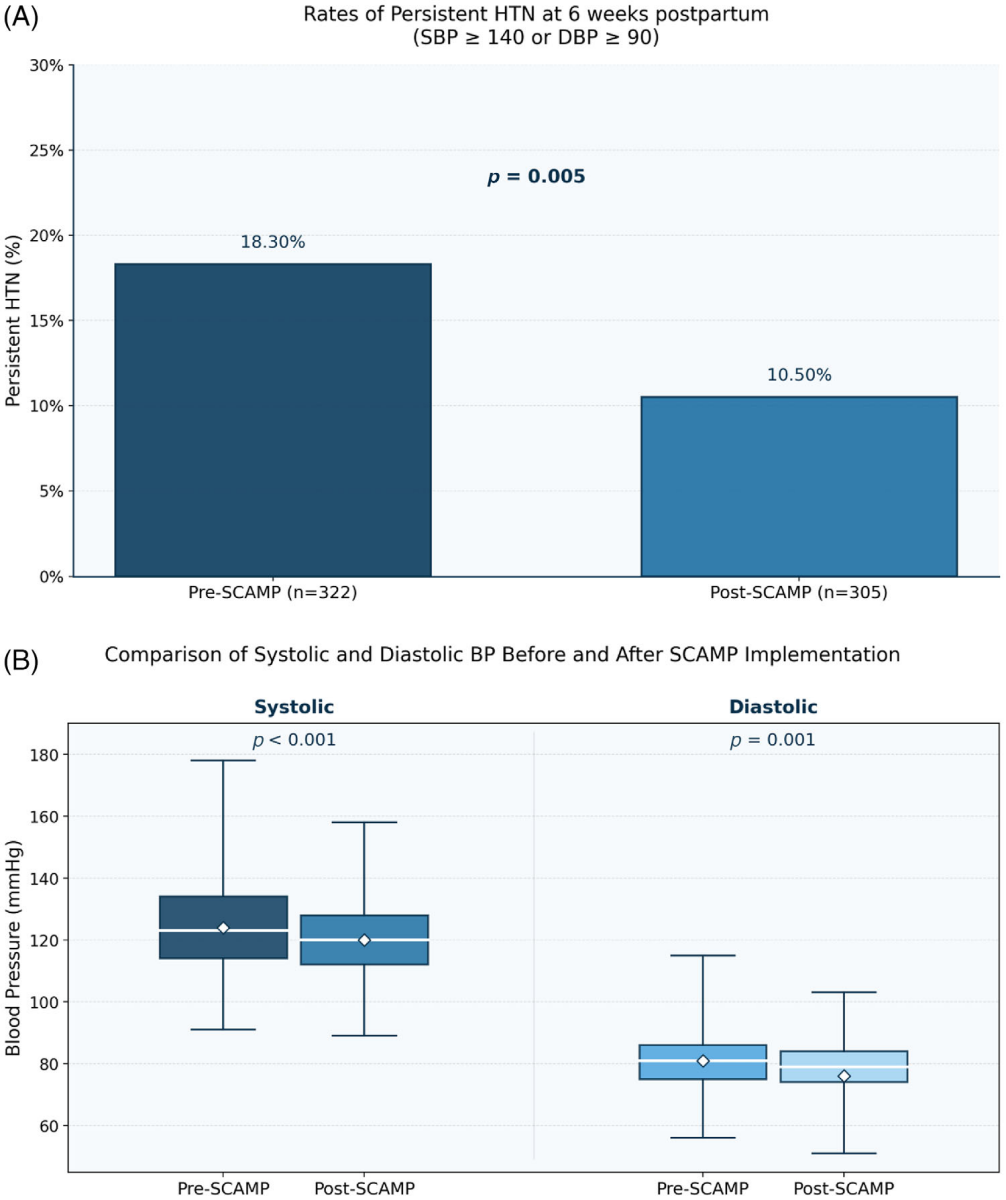
(A) Comparison of rates of HTN at 6 weeks postpartum. (B) Median and interquartile range (IQR) of systolic and diastolic BP (SBP and DBP) at 6 weeks postpartum before and after SCAMP implementation. Standard deviation (SD) is noted with a diagonal box. HDP, hypertensive disorders of pregnancy; HTN, hypertension; SCAMP, standardized clinical assessment and management plan.

In secondary outcomes, mean systolic BP at the 6‐week PP visit was significantly lower in the SCAMP group compared with controls (120 ± 12 vs. 124 ± 14 mmHg, *p* < 0.001). Similarly, mean DBP was lower in the SCAMP group (76 ± 8 vs. 81 ± 8 mmHg, *p* < 0.001) (Table 2).

BP ascertainment patterns also differed significantly between groups. A higher proportion of patients in the SCAMP group had any BP recorded prior to their 6‐week appointment compared with controls (87.9% vs. 62.1%; aOR, 4.35; 95% CI, 2.86–6.62; *p* < 0.001). Additionally, the median time from discharge to first BP ascertainment was markedly shorter in the SCAMP group (1‐day, interquartile range [IQR] 1–6 days vs 13 days, IQR 3–41 days, *p* < 0.001).

There were no differences between groups in the proportion of patients on anti‐HTN medications at 6 weeks PP (*p* = 0.52) or who continued anti‐HTN medications beyond 6 weeks PP (*p* = 0.78). However, a lower proportion of patients in the SCAMP group were seen by primary care or cardiology during the PP period compared with controls (49.8% vs 58.1%; aOR, 0.72; 95% CI, 0.52–0.99; *p* = 0.038).

In our subanalysis of patients with hypertensive diseases of pregnancy and excluding those with only chronic hypertension, we found similar outcomes as above (Table S1). In the SCAMP cohort compared to controls, there were significantly lower rates of hypertension at 6 weeks PP (6.2% vs 16.2%, *p* < 0.001) and lower average systolic (118 ± 12 mmHg vs 123 ± 14 mmHg, *p* < 0.001) and DBP (78 ± 7 mmHg vs 80 ± 9 mmHg, *p* < 0.001). The SCAMP cohort had higher rates of having any BP recorded prior to 6 weeks PP (86.4% vs. 62.8%, *p* < 0.001) and lower rates of PP readmission (1.2% vs. 6.9%, *p* = 0.001).

## 4 DISCUSSION

### 4.1 Principal findings

In this secondary analysis of a prospective study using historical controls, we found that implementation of a SCAMP for PP HTN was associated with a significant reduction in HTN at 6 weeks PP, as well as with lower mean SBP and DBP values at the 6‐week visit. The post‐intervention group also had higher BP ascertainment rates prior to their PP follow‐up.

### 4.2 Results/ clinical implications

The improved BP ascertainment patterns in the SCAMP group showed that nearly 88% of patients in the SCAMP group had BP recorded prior to their 6‐week appointment, with a median of 1 day from discharge to first documented BP. This represents a substantial improvement over traditional care patterns, where BP checks typically occur 1–2 weeks after discharge [16]. This difference is an expected consequence of cuff provision and remote monitoring enrollment and is presented as evidence of protocol fidelity rather than as an independent outcome. It does, however, describe the mechanism by which the SCAMP enabled medication titration earlier in the PP course than usual care permits.

Many patients with HDP will have HTN BPs following discharge, with a significant number requiring anti‐HTN treatment [24, 25, 26, 27, 28]. Beyond the immediate PP period, patients with HDP have a 2 to 4‐fold increased risk of developing chronic HTN [29, 30, 31, 32, 33, 34]. In nonpregnant individuals, intensive systolic BP control < 120 is associated with significantly lower CV events and overall mortality [35]. In PP individuals, elevated BP at the 6‐week PP visit has been associated with chronic HTN years later [36]. However, significant variability exists in PP HTN management and BP goals [37]. Confirming whether protocol‐driven PP management alters the trajectory toward chronic HTN or further CV risk in a real‐world, unselected population will require follow‐up measured off therapy at 6 to 12 months PP and beyond.

Interestingly, the SCAMP group had lower rates of referral to or visits with primary care or cardiology during the PP period (49.8% vs 58.1%). This may reflect better BP control in the SCAMP group, reducing perceived need for specialist referral. It may also suggest that the structured SCAMP follow‐up provided sufficient BP management within the immediate PP period. We recommend all patients with HTN have follow up with their primary care providers with escalation to cardiology as clinically indicated, such as for preeclampsia with severe features or persistent need for anti‐HTN medications. Further investigation into care transitions and patient outcomes after SCAMP enrollment would help clarify the optimal timing and pathway for ongoing CV care in this population.

### 4.3 Strengths and limitations

Strengths of this study include the large sample size within a single hospital system, allowing for consistent application of the SCAMP protocol and comparison groups that were similar in most baseline characteristics. The secondary analysis design builds on a well‐characterized primary cohort study with detailed BP and outcome data. The use of a SCAMP model, which provides clinical guidance while allowing for physician judgment and protocol adaptation, represents a pragmatic approach that may be more feasible to implement than rigid protocols in diverse clinical settings. When removing patients with only chronic hypertension from the cohort, we still found similar findings of lower rates of HTN at 6 weeks PP in the SCAMP cohort, demonstrating the impact of SCAMP regardless of specific hypertension diagnosis.

Our study has several limitations. As a secondary analysis, this study was not powered a priori for the primary outcome. The use of historical controls may introduce selection bias or temporal trends that could affect the results, though baseline characteristics were generally similar between groups. An important limitation is that 153 patients (19.6%) from the primary cohort were excluded from this analysis due to lack of documented BP at a 6‐week PP visit. These patients may differ systematically from those who attended follow‐up visits, potentially introducing selection bias. Patients without 6‐week visit documentation may have had worse BP control, better BP control requiring no follow‐up, or barriers to care access. This limitation is common in PP research, as PP visit attendance rates are historically low, with studies showing that up to 40% of women do not attend recommended PP visits [38]. However, our exclusion rate of 19.6% is lower than many PP studies, suggesting reasonable follow‐up rates in both groups.

Our primary outcome was ascertained at 6 weeks PP, coinciding with the conclusion of the remote monitoring program, and therefore reflects BP measured during rather than after the intervention period. Although anti‐HTN use did not differ between cohorts at this time point, residual differences in treatment intensity cannot be excluded. BP measured off therapy at 6 to 12 months PP would be required to determine whether early intensive management alters the trajectory toward chronic HTN, and our analysis cannot address that question.

The analysis was performed at a single tertiary care center with specific patient demographics and resources, which may limit generalizability to community settings or institutions with different patient populations, resources, or EMR capabilities. The remote BP monitoring system required specific technical infrastructure and dedicated staff for monitoring and responding to alerts, which may not be available at all institutions. However, the core principles of aggressive inpatient BP management and early outpatient follow‐up could potentially be adapted to other settings using available resources.

### 4.4 Research implications

As this is a secondary analysis examining outcomes at 6 weeks PP, longer‐term follow‐up data are needed to assess whether the observed improvements in BP control are sustained and translate to reduced risk of chronic HTN and CV disease. Future studies should examine BP control and CV outcomes at 6 months, 1 year, and beyond to determine the long‐term impact of aggressive early PP BP management.

Given our findings, we suggest that strategies to improve PP BP control should focus on both inpatient management and early outpatient surveillance. The combination of lower BP targets before discharge (BP < 140/90 mmHg) and immediate initiation of remote BP monitoring upon discharge appears to be effective in reducing HTN at 6 weeks. Future quality improvement initiatives could consider implementing similar bundled approaches, potentially incorporating patient education, dedicated nursing support, and systematic protocols for BP management and follow‐up.

## 5 CONCLUSION

Implementation of a PP HTN SCAMP with intensive inpatient BP management and structured outpatient remote monitoring was associated with reduced HTN at 6 weeks PP, along with earlier BP ascertainment and lower rates of specialist referral. These findings suggest that proactive BP management in the immediate PP period may have lasting benefits beyond preventing readmissions. Additional research is needed to determine whether these improvements translate to reduced long‐term CV risk in women with HDP.

## CONFLICT OF INTEREST STATEMENT

The authors declare no conflicts of interest.

## FUNDING INFORMATION

The authors received no specific funding for this work.

## ETHICS STATEMENT

All work completed was compliant with our institutional review board.

## Supporting information



Supporting information
