# Supplementary material for: Standardized postpartum blood pressure protocol and lower rates of hypertension at 6 weeks postpartum
Source: Pregnancy (Hoboken). 2026 Oct 8;2(6):e70477. doi: 10.1002/pmf2.70477 (PMC13650410; doi:10.1002/pmf2.70477)
Supplement: Supplementary file 1 [file PMF2-2-e70477-s001.docx]

**Supplemental Table 1. Six-Week Postpartum Hypertension Outcomes Between Groups with Only Patients who Developed Hypertensive Disease of Pregnancy**

| **Six-Week Postpartum Outcomes** | **SCAMP (n=305)** | **Controls (n=322)** | **p-value** |  |
| --- | --- | --- | --- | --- |
| HTN at 6 weeks PP (SBP≥140 or DBP≥90) | 16 (6.2%) | 40 (16.2%) | **<0.001** |  |
| Average systolic BP at 6 weeks PP (mmHg, mean ± SD) | 118 ± 12 | 123 ± 14 | **<0.001** |  |
| Average diastolic BP at 6 weeks PP (mmHg, mean ± SD) | 78 ± 7 | 80 ± 9 | **<0.001** |  |
| Any BP recorded prior to 6-week appointment | 222 (86.4%) | 155 (62.8%) | **<0.001** |  |
| Number of days after discharge of first BP ascertainment (days, median, IQR) | 1 (1,6) | 14 (3, 41) | **<0.001** |  |
| Postpartum readmission | 3 (1.2%) | 17 (6.9%) | **0.001** |  |
| On anti-HTN medication at 6 weeks PP | 36 (14.0%) | 37 (15.0%) | 0.76 |  |
| Continued on anti-HTN medication past 6 weeks PP | 31 (12.1%) | 29 (11.7%) | 0.91 |  |
| Seen by primary care or cardiology PP | 122 (47.5%) | 138 (56.1%0 | 0.053 |  |
| Data presented as n (%) unless otherwise indicated | | | | |
| Abbreviations: SCAMP, standardized clinical assessment and management plan; HTN, hypertension; SBP, systolic blood pressure; DBP, diastolic blood pressure; BP, blood pressure; IQR, interquartile range | | | | |
| P-value calculated for t-test, Mann-Whitney U, and chi-square as appropriate to compare groups. P-value significance was set at <0.05 and significant values are bolded. | | | | |
| Patients were included as seen by primary care or cardiology if they attended an outpatient visit, either virtually or in-person, within 12 weeks postpartum. | | | | |
